# Phenazine-1-Carboxylic Acid (PCA), Produced for the First Time as an Antifungal Metabolite by *Truncatella angustata*, a Causal Agent of Grapevine Trunk Diseases (GTDs) in Iran

**DOI:** 10.1021/acs.jafc.1c03877

**Published:** 2021-10-08

**Authors:** Alessio Cimmino, Zeinab Bahmani, Stefany Castaldi, Marco Masi, Rachele Isticato, Jafar Abdollahzadeh, Jahanshir Amini, Antonio Evidente

**Affiliations:** † Department of Chemical Sciences, University of Naples Federico II, Complesso Universitario Monte S. Angelo, Via Cintia 4, 80126 Napoli, Italy; ‡ Department of Plant Protection, Agriculture Faculty, University of Kurdistan, P.O. Box 416 Sanandaj, Iran; § Department of Biology, University of Naples Federico II, Complesso Universitario Monte S. Angelo, Via Cintia 4, 80126 Napoli, Italy

**Keywords:** Truncatella angustata, phenazine-1-carboxylic acid (PCA), phenazine, antifungal activity, biological control

## Abstract

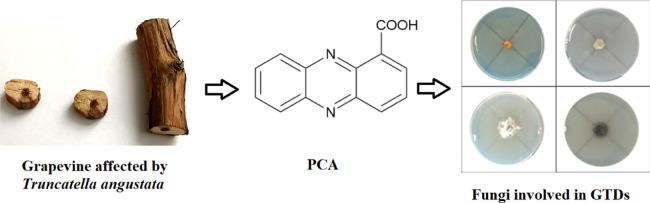

The phytopathogenic fungus *Truncatella angustata*, associated with grapevine trunk diseases (GTDs) in Iran, produces the well-known secondary metabolite isocoumumarin (+)-6-hyroxyramulosin and surprisingly also phenazine-1-carboxylic acid (PCA). PCA, identified by spectroscopic (essentially ^1^H NMR and ESI MS) spectra, is a bacterial metabolite well known for its antifungal activity and was found for the first time in *T. angustata* culture filtrates. The antifungal activity of PCA was assayed against four different fungi responsible for GTDs, *Phaeoacremonium minimum*, *Phaeoacremonium italicum*, *Fomitiporia mediterranea*, involved in grapevine esca disease, and *Neofusicoccum parvum*, responsible for Botryosphaeria dieback. The activity was compared with that of the known commercial fungicide, pentachloronitrobenzene, and the close phenazine. PCA and phenazine exhibited strong antifungal activity against all phytopathogenic fungi, inhibiting the fungal growth by about 90–100% and 80–100%, respectively. These results suggested that *T. angustata* could use PCA to compete with other phytopathogenic fungi that attack grapevine and thus PCA could be proposed as a biofungicide against the fungi responsible for grapevine esca and Botryosphaeria dieback diseases.

## Introduction

The economic importance of grapevine (*Vitis vinifera* L.) has grown exponentially in recent years and many efforts have been made to increase its production yield and the organoleptic qualities of wine.^1,2^ Unfortunately, grapevine can be affected by several biotic stress agents that are considered a major threat to the economic sustainability of viticulture.^2,3^ Among these, pathogenic fungi cause significant losses by inducing severe diseases in different plant organs. They are able to produce toxic metabolites belonging to several classes of naturally occurring compounds whose role in the plant–pathogen interaction is under study.^3^ However, the most important grapevine diseases are related to the woody tissues, i.e., trunk and cordons, and are called grapevine trunk diseases (GTDs). There are no effective methods for the control of GTDs and the prevention of infections is mainly based on the application of chemical pesticides.^4^ For these reasons, environmentally friendly alternatives for controlling GTDs are urgently needed and could be based on the use of natural fungicides.

*Truncatella angustata* was recently reported as one of the causal agents of GTDs in Iran and was shown to produce (+)-6-hydroxyramulosin, a well-known phytotoxin.^5^ Surprisingly, *T. angustata* also produced a yellow compound, which showed antifungal activity against some fungi involved in GTDs, suggesting a potential role in the microbial interaction in the diseased grapevine.

Thus, the aims of this manuscript were the isolation and chemical and biological characterization of this metabolite. This was identified as phenazine-1-carboxylic acid (PCA), a compound frequently isolated from *Pseudomonas* spp. and well known for its antifungal activity and potential application in agriculture as a potential fungicide to control phytopathogens that infect the agricultural plants with high world market value.^6,7^

Thus, this manuscript reports the isolation of phenazine-1-carboxylic acid for the first time from the culture filtrates of the phytopathogenic fungus *T. angustata* and its involvement in GTDs in Iran. Its role in completely inhibiting the growth of other fungi competing in the same environment has also been discussed.

## Materials and Methods

### General Experimental Procedures

^1^H NMR spectra were recorded at 400 MHz, respectively, in CDCl_3_ on a Bruker spectrometer (Karleshrue, Germany). The same solvent was used as an internal standard. Electrospray ionization (ESI) mass spectra and liquid chromatography LC/MS analyses were performed using the LC/MS time-of-flight (TOF) system Agilent 6230B (Agilent Technologies, Milan, Italy) and high-performance liquid chromatography (HPLC) 1260 Infinity. The HPLC separations were performed with a Phenomenex (Bologna, Italy) LUNA (C_18_ (2) 5 μm 150 × 4.6 mm). Analytical and preparative thin-layer chromatography (TLC) was performed on silica gel plates (Merck, Kieselgel 60, F_254_, 0.25 and 0.5 mm, respectively) or on reverse phase (Whatman, C_18_ F_254_, 0.20 mm) plates (Merck, Darmstadt, Germany), and the compounds were visualized by exposure to UV light and/or iodine vapors CC: silica gel (Merck, Kieselgel 60, 0.063–0.200 mm). The sample of standard phenazine was purchased from Sigma-Aldrich (Milan, Italy).

### Fungal Strains

The strain of *T. angustata* (CJAZBSRK1) used in this study was obtained from a vineyard showing symptoms of grapevine trunk diseases including decline and vascular discoloration and necrosis, located in Dinavar district, Sahneh, Kermanshah Province, Iran. The fungus was purified using a single-spore technique. DNA extraction, PCR, and maximum parsimony analysis were carried out as described by Abdollahzadeh et al. (2009).^8^ For the identification of *T. angustata* ITS region of ribosomal DNA was amplified. To confirm its pathogenicity under greenhouse conditions (22–28 °C), Koch’s postulates were followed. *T. angustata* strain (CJAZBSRK1) was stored on potato dextrose agar (PDA) at 4–8 °C in the fungal collection of the Department of Plant Protection, University of Kurdistan, Iran. The fungal strains of *Phaeoacremonium minimum*, *Fomitiporia mediterranea*, and *Neofusicoccum parvum* were supplied by Prof. Laura Mugnai of the Department of Science and Technology Agriculture, Food, Environmental and Forestry (DAGRI), Sec. Pathology and Entomology, University of Florence, Florence, Italy. The strain of *Phaeoacremonium italicum* was supplied by Prof. Antonia Carlucci of the Department of Agricultural Sciences, Food, Natural Resources and Engineering, University of Foggia, Foggia, Italy.

### Production, Extraction, and Purification of PCA

For metabolite production, *T. angustata* was inoculated and grown in a stationary culture (final volume 5 L) of the Potato Dextrose Broth (PDB) as previously reported.^5^ The lyophilized culture filtrates (5 L) of *T. angustata* were dissolved in 1/10 of the initial volume (pH 6) and extracted with EtOAc as recently reported.^5^ The organic extracts were combined, dried (Na_2_SO_4_), and evaporated under reduced pressure, giving a corresponding residue of 330 mg. This latter was purified by silica gel column chromatography and eluted with CHCl_3_/*i*-PrOH (9/1, v/v) to (7/3, v/v) yielding seven homogeneous fraction groups. The residue of fraction 2 (10.0 mg) was further purified by TLC on the reverse phase eluted with CH_3_CN/H_2_O (7:3, v/v), yielding a yellow amorphous solid identified as phenazine-1-carboxylic acid (PCA) (**1**, R*f* 0.48, 6.0 mg).

### Antifungal Assays

The phytopathogenic fungi *P. minimum* (PV.FI.A.188), *P. italicum* (Pm 45), *F. mediterranea* (PV.FI.A.132), and *N. parvum* (PV.FI.A.41) were grown separately on PDA in Petri dishes at 25 °C ± 1 for 7/8 days in darkness. The *in vitro* antifungal bioassays were carried out according to the method previously described by Puopolo et al. (2013)^9^ with some modifications. PCA and phenazine dissolved in MeOH and pentachloronitrobenzene (PCNB) (Sigma-Aldrich, Saint Louis, MO) dissolved in toluene were placed on the opposite four sides of the plates 1.5 cm away from the fungal disk at a final concentration of 25 μg/μL. MeOH and toluene were used in the same conditions as negative controls. The plates were incubated at 25 °C ± 1 for 7/8 days and examined for zones of inhibition of grown colonies. Plates containing the fungal plugs alone were used as control. The experiments were performed in triplicate. The percentage of inhibition of the fungal growth was calculated using the following formula
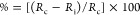
where *R*_c_ is the radial growth of the test pathogen in the control plates (mm) and *R*_i_ is the radial growth of the test pathogen in the test plates (mm). The results were analyzed by analysis of variance (ANOVA) using Tukey’s test.

## Results and Discussion

**Figure 1:**
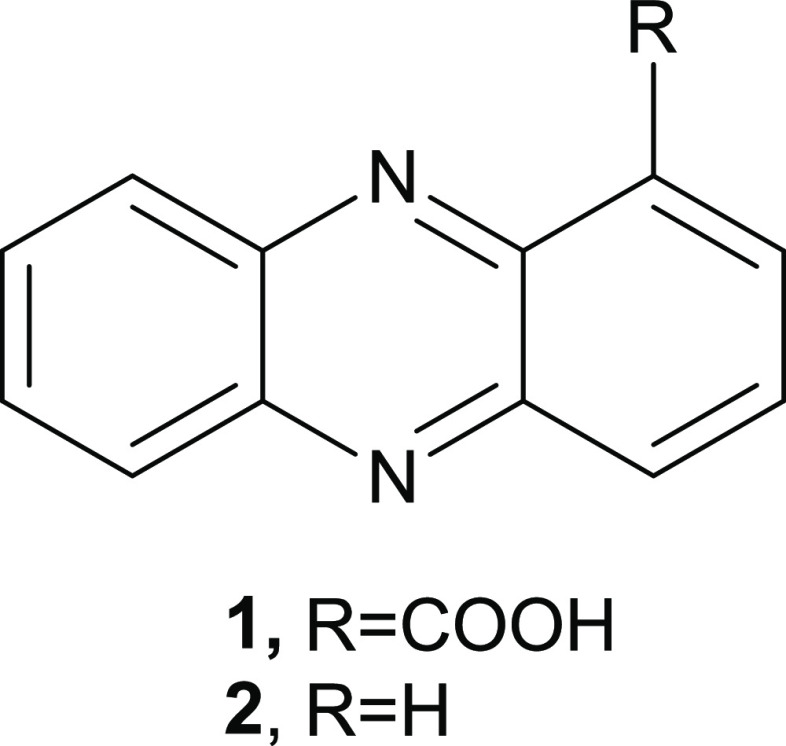
Structure of phenazine-1-carboxylic acid and phenazine (**1** and **2**).

PCA (**1**, Figure 1) was isolated from the organic extract of *T. angustata* culture filtrates and identified by comparing its ^1^H NMR and ESI MS with those previously reported^10^ and those of an authentic sample previously isolated from *Pseudomonas chlorophasis* subsp. *aureofaciens* strain M71.^10^

This *Pseudomonas* strain produced compound **1** together with 2-hydroxyphenazine and was proposed as a potential agent for the biocontrol of *Seiridium cardinale*, the fungus responsible for the bark canker of Italian cypress (*Cupressus sempervirens* L.).^10^ When **1** was applied *in vitro* against *S. cardinale*, the canker size was reduced, indicating that it is directly involved in the control of the pathogen by *P. chlororaphis* subsp. *aureofaciens* strain M71.^10^ This result was also confirmed by field experiments.^11^ Studies were also carried out to estimate the spectrum of the activity of PCA, 2-hydroxyphenazine, and four semisynthetic PCA derivatives against a group of pathogenic fungi of agricultural and forest plants by an agar plate bioassay. PCA was active against most of the plant pathogens tested, showing that the carboxyl group is a structural feature important for the antifungal activity.^9^

PCA belongs to the well-known synthetic and natural phenazine group, which includes more than 100 different compounds of natural origin and over 6000 synthetic compounds. Many of them were studied for their potential application in different fields such as in medicine as anticancer agents^12^ and against cystic fibrosis.^13,14^ It could also be used in other biotechnological applications as fluorescent material for the advancement of modern science and technology.^15^

Rarely, PCA was isolated from fungi. In fact, **1** and its amide were previously isolated from *Nigrospora oryzae* obtained from the medicinal plant *Coccinia grandis*, and the carboxyamide showed antifungal activity against the plant pathogen *Cladosporium cladosporioides*.^16^

Recently, **1** was reported as an antimicrobial metabolite isolated from the sea anemone-derived fungus *Emericella* sp., showing antifungal activity against *Phytophthora capsici*, *Gibberella zeae*, and *Verticillium dahliae*.^17^

In addition, some of the authors isolated phenazine from *Pseudomonas fluorescens* 9,^18^ a strain isolated in Argentina and proposed for the control of *Macrophomina phaseolina*, which infects soybean and more than 500 plant species belonging to more than 100 families, causing dry root and stem rot, known as charcoal rot (CR).^19^ Thus, PCA (**1**), phenazine (**2**, Figure 1), 2-hydroxyphenazine, and some mono and dinitrophenazine derivatives, prepared by nitration of **2**, were assayed against *M. phaseolina* and also against two other destructive fungi infecting soybeans such as *Cercospora nicotianae* and *Colletotrichum truncatum*. Phenazine and PCA showed the same strong antifungal activity against the three pathogens while 2-hydroxyphenazine, assayed only against *M. phaseolina*, was inactive. Finally, all nitrophenazine derivatives not showed antifungal activity against *M. phaseolina* while exhibited antifungal activity against *C. nicotianae* and *C. truncatum*. In particular, in *C. nicotianae*, they showed a slightly reduced activity than in **1** and **2**, while on *C. truncatum*, the inhibition effect of these derivatives appeared to be significantly reduced. Thus, probably the activity is also dependent on the sensitivity of the fungal species.^18^

Consequently, PCA and phenazine can be evaluated for their potential antifungal activity against the fungi involved in GTDs.

**Figure 2:**
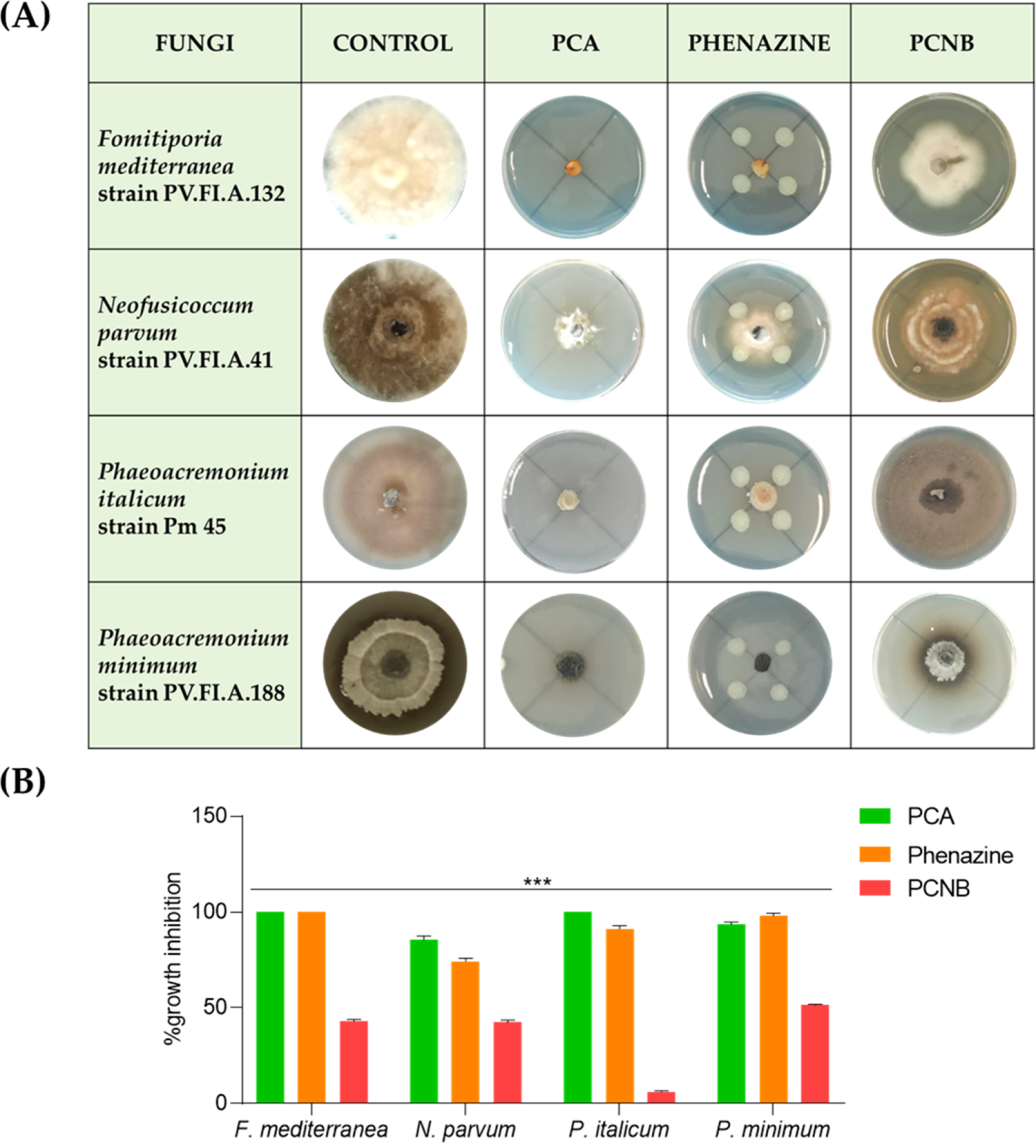
*In vitro* antifungal activity of PCA: phenazine-1-carboxylic acid; phenazine and PCNB: pentachloronitrobenze tested at 25 μg/μL. (A) Representative photographs of the antifungal assay for *in vitro* inhibition of mycelial growth of *P. minimum*, *P. italicum*, *F. mediterranea*, and *N. parvum*, (B) Inhibition of fungal growth by PCA, phenazine, and PCNB reported as the percentage reduction in the diameter of the fungal mycelia in the treated plate compared to that in the control plate. Data are presented as mean ± standard deviation (*n* = 3) compared to control fungi grown alone. For comparative analysis of groups of data, one-way ANOVA was used and *p* values are presented in the figure: ***: extremely significant <0.001.

PCA (**1**) compared to phenazine (**2**) and the commercial fungicide pentachloronitrobenzene (PCNB) were assayed against some fungi involved in GTDs as *P. minimum*, *P. italicum*, *F. mediterranea* involved in esca disease^3,20^ and *Na. parvum*, one of the causal agents of Botryosphaeria dieback.^3^ As shown in Figure 2, the PCA exhibited strong antifungal activity against all phytopathogenic fungi, inhibiting the fungal growth by about 90–100% when spot-inoculated at a final concentration of 25 μg/μL. Similarly, phenazine has shown strong growth-inhibiting activity in all fungi, respectively, by 80–100% when tested at the same concentration of PCA. A different result was obtained with the commercial fungicide PCNB. At the same concentration (25 μg/μL) used for PCA and phenazine, the PCNB showed a lower fungal growth inhibition activity by about 10–48%.

In conclusion, this manuscript reports for the first time the isolation of phenazine-1-carboxylic acid from a phytopathogenic fungus as *T. angustata*, a causal agent of GTDs in Iran. Its isolation as a fungal metabolite is very rare considering that only three other fungi, two of which were isolated from marine organisms, have been reported as PCA producers. The production of PCA by *T. angustata* is probably due to inhibit the growth of other pathogenic fungi that could attack grapevine. This hypothesis has been confirmed by the results of the bioassays carried out against some fungi responsible for GTDs. In fact, the PCA has shown strong antifungal activity inhibiting the fungal growth of *P. minimum*, *P. italicum*, *F. mediterranea*, and *N. parvum* of about 90–100%. Thus, PCA could be proposed as a biofungicide against the fungi responsible for grapevine esca and Botryosphaeria dieback diseases.
